# Correction: VPS13D bridges the ER to mitochondria and peroxisomes via Miro

**DOI:** 10.1083/jcb.20201000405052021c

**Published:** 2021-06-22

**Authors:** Andrés Guillén-Samander, Marianna Leonzino, Michael G. Hanna, Ni Tang, Hongying Shen, Pietro De Camilli

Vol. 220, No. 5 | 10.1083/jcb.202010004 | April 23, 2021

The authors contacted the editors shortly after publication to make the following changes in order to meet the requirements set by the funding agency Aligning Science Across Parkinson’s (ASAP) Collaborative Research Network: (1) add the Aligning Science Across Parkinson’s (ASAP) Collaborative Research Network to the affiliations; (2) correct the Acknowledgments section; (3) change the license to CC-BY; and (4) add links to deposited data and protocols to meet requirements. In addition, (5) the authors noticed a mistake in the legend of Fig. 2 C. The article has been corrected as follows:

1. Affiliation #5 was added as shown below:

Andrés Guillén-Samander^1,5^, Marianna Leonzino^1,5^, Michael G. Hanna IV^1^, Ni Tang^1,5^, Hongying Shen^3,4^, and Pietro De Camilli^1,2,5^

^1^Departments of Neuroscience and of Cell Biology, Howard Hughes Medical Institute, Program in Cellular Neuroscience, Neurodegeneration and Repair, Yale University School of Medicine, New Haven, CT

^2^Kavli Institute for Neuroscience, Yale University School of Medicine, New Haven, CT

^3^Department of Cellular and Molecular Physiology, Yale University School of Medicine, New Haven, CT

^4^Systems Biology Institute, Yale West Campus, West Haven, CT

^5^Aligning Science Across Parkinson’s (ASAP) Collaborative Research Network, Chevy Chase, MD

2. The following text was added to the Acknowledgments:

“The study was also funded by the joint efforts of the Michael J. Fox Foundation for Parkinson’s Research (MJFF) and the Aligning Science Across Parkinson’s (ASAP) initiative. MJFF administers the grant ASAP-000580 on behalf of ASAP and itself. For the purpose of open access, the author has applied a CC-BY public copyright license to the Author Accepted Manuscript (AAM) version arising from this submission.”

3. The article is now available under a CC-BY 4.0 license.

4. The protocols were uploaded to the public database protocols.io and are now cited in the “DNA plasmids,” “Cell culture and transfection,” “Neuronal cultures,” “Microscopy,” and “Image processing, analysis, and statistics” sections of the Materials and methods.

5. The corrected legend of Fig. 2 C appears below:

“Quantification of VPS13D^EGFP enrichment at mitochondria in control conditions and upon Miro knockdown or overexpression. The signal from mito-BFP was first used to generate a mitochondrial mask and a mask profiling a thin (1-pixel wide) cytosolic area surrounding mitochondria; the intensity from EGFP was then measured within each of these masks, and, for each cell analyzed, the ratio between these two measurements was plotted on the graph. Number of cells analyzed for scrambled (Scr) RNAi, Miro RNAi, and mCh-Miro1 conditions are 204, 204, and 96, respectively. ****, P < 0.0001 (Welch’s corrected ANOVA with Games-Howell’s post hoc test).”

“Games-Howell’s post hoc test” replaced “Bonferroni post hoc test.” This change does not impact the data.

The previously published information appears only in print and in PDF versions downloaded on or before June 17, 2021.

